# The effect of endometrial thickness and pattern measured by ultrasonography on pregnancy outcomes during IVF-ET cycles

**DOI:** 10.1186/1477-7827-10-100

**Published:** 2012-11-28

**Authors:** Jing Zhao, Qiong Zhang, Yanping Li

**Affiliations:** 1 Reproductive Medicine Center, Xiangya Hospital, Central South University, Changsha, Hunan, China

**Keywords:** Endometrial thickness, Endometrial pattern, IVF-ET, Implantation, Pregnancy

## Abstract

**Background:**

To study the effect of endometrial thickness and pattern measured using ultrasound upon pregnancy outcomes in patients undergoing IVF-ET.

**Method:**

One thousand nine hundred thirty-three women undergoing IVF treatment participated in the study. We assessed and recorded endometrial patterns and thickness on the day of human chorionic gonadotropin (hCG) administration. Receiver operator curves (ROC) were used to determine the predictive accuracy of endometrial thickness. Cycles were divided into 3 groups depending on the thickness (group 1: ≤ 7 mm; group 2: > 7 mm to ≤ 14 mm; group 3: > 14 mm). Each group was subdivided into three groups according to the endometrial pattern as follows: pattern A (a triple-line pattern consisting of a central hyperechoic line surround by two hypoechoic layers); pattern B (an intermediate isoechogenic pattern with the same reflectivity as the surrounding myometrium and a poorly defined central echogenic line); and pattern C (homogenous, hyperechogenic endometrium). Clinical outcomes such as implantation and clinical pregnancy rates were analyzed.

**Results:**

The endometrial thickness predicts pregnancy outcome with high sensitivity and specificity. The cutoff value was 9 mm. The implantation rate and clinical pregnancy rate in group 3 were 39.1% and 63.5%, respectively, which were significantly higher than those in group 2 (33.8% and 52.1%, respectively) and group 1 (13% and 25.5%, respectively). Among those with Pattern A, the implantation rate and clinical pregnancy rate were 35.3% and 55.2%, respectively, which were significantly higher than among women with Pattern B (32.1% and 50.9%, respectively) and Pattern C (23.4% and 37.4%, respectively). In groups 1 and 3, clinical pregnancy and implantation rates did not show any significant differences between different endometrial patterns (P > 0.05), whereas in group 2, the clinical pregnancy rate and implantation rate in women with pattern A were significantly higher than those with pattern B or C (P < 0.05).

**Conclusions:**

Endometrial thickness and pattern independently affect pregnant outcomes. Combined endometrial thickness and pattern could not predict the outcome of IVF-ET when endometrial thickness was < 7 mm or >14 mm, while a triple-line pattern with a moderate endometrial thickness appeared to be associated with a good clinical outcome.

## Background

The success of in vitro fertilization and embryo transfer (IVF-ET) cycles depends mainly on embryo quality and uterine receptivity [1]. With respect to uterine receptivity, evaluation of endometrial receptivity continues to be a challenge in assisted reproductive technology (ART). Ultrasonographic examination has been routinely performed for evaluation of the endometrium in ART treatment because it allows accurate and noninvasive evaluation.

Although many studies have implicated endometrial thickness and pattern as prognostic parameters for successful outcomes in IVF-ET, there is still no consensus on whether the endometrial ultrasound characteristics can predict the pregnancy outcome. Many studies have shown a correlation between endometrial thickness or a certain type of echogenic pattern and uterine receptivity [2-10]. Some studies have suggested a minimal thickness for a successful pregnancy to occur, while others have reported adverse effects of increased endometrial thickness above which pregnancy is unlikely to occur [6,11,12]. In contrast, others have failed to demonstrate a relationship between endometrial thickness, pattern, and pregnancy and implantation rates [13-17]. Furthermore, few studies have combined endometrial thickness and pattern to predict the outcome of IVF-ET. The aim of our study was to evaluate the endometrial characteristics on the day of hCG administration. In particular, we aimed to assess the correlation between endometrial thickness and pattern (individually and together) and IVF outcome.

## Methods

### Patient recruitment and counseling

The study was reviewed and approved by the Institutional Review Board and the Ethics Committee of Xiangya Hospital, Changsha, China. The study was conducted in accordance with the Declaration of Helsinki, as revised in 1983. We conducted a retrospective cohort study of 1933 consecutive infertile patients. Briefly, patients underwent fresh IVF-ET between January of 2009 and May of 2011 at the Reproductive Medicine Center of Xiangya Hospital Central South University (Changsha, China). Exclusion criteria included the following: the presence of a known endometrial polyp or uterine anomaly, an insemination method other than IVF, and cycles using donor oocytes or cryopreserved embryos. Patients underwent no therapeutic interventions except routine procedures.

### Ovulation induction and IVF-ET precedures

The choice of stimulation protocol was individual and was based on the patient’s age, diagnosis, reproductive history and ovarian response, and coexisting medical conditions. When the serum estradiol concentration (E2) level was ≤50 pg/ml, and the longest follicle diameter was <10 mm without ovarian cysts, controlled ovarian hyperstimulation (COH) was performed. COH was achieved with administration of gonadotrophin, including follicle stimulating hormone (FSH) and/or human menopausal gonadotrophin (hMG). The initial dosage of gonadotrophin ranged from 150 to 450 IU, depending on the basal FSH level, antral follicular count (AFC), and maternal age. When at least two follicles were ≥18 mm in diameter and when serum E2 level was within the acceptable range for the number of mature follicles present, 10000 IU of hCG was administered. Oocyte retrieval was performed 36 hours after the administration of hCG and followed by conventional IVF. Up to three embryos were transferred 72 hours after oocyte collection. The luteal phase was supported using a daily intramuscular injection of 80 mg of progesterone in oil. Biochemical pregnancies were considered as failure to conceive. Clinical pregnancy was defined as identification of a gestational sac 4–5 weeks after embryo transfer.

### Ultrasound measurement

Measurement of endometrial thickness and pattern was performed 11–12 hours before the hCG injection by transvaginal 8 MHz ultrasonography with Doppler Ultrasound (Mindray DC-6 Expert, Shenzhen, China) after patients had rested for at least 15 minutes and completely emptied their bladders. Endometrial thickness was measured in a median longitudinal plane of the uterus as the maximum distance between the endometrial-myometrial interface of the anterior to the posterior wall of the uterus. All cycles were divided into the following three group depending on the thickness: group 1: ≤ 7 mm; group 2: > 7 mm to ≤ 14 mm; group 3: > 14 mm. Endometrial pattern was classified as pattern A (a triple-line pattern consisting of a central hyperechoic line surrounded by two hypoechoic layers), pattern B (an intermediate isoechogenic pattern with the same reflectivity as the surrounding myometrium and a poorly defined central echogenic line), or pattern C (homogenous, hyperechogenic endometrium). Endometrial thickness groups were subdivided into three endometrial types.

### Statistical analysis

Continuous data are expressed as the mean ± SD values or as median and range according to the distribution and were analyzed with Student’s *t*-test. Categorical data were presented as counts, and the statistical comparison of percentage was carried out with the chi-square test. Statistical analysis was performed with SPSS (Statistical Package for Social Science, SPSS Inc, Chicago, IL, USA) version 16.00. P < 0.05 was considered statistically significant.

## Results

The clinical pregnancy rate was 52.3%, and the implantation rate was 33.2%. Patients ranged in age from 21 to 47 years, and endometrial thickness on the day of hCG administration ranged from 4.8 mm to 28.02 mm. Other demographic date, such as basal FSH, duration of infertility, and number of embryos transferred are summarized in Table 1.

**Table 1:** Characteristics of the study group (n = 1933)

**Characteristic**	**Mean ± SD**
Age(years)	31.18 +/− 4.62
Infertility(years)	4.84 +/− 3.34
Baseline FSH(IU/L)	6.26 +/− 2.90
Length of stimulation(days)	11.12 +/− 2.32
Total dose of Gn(IU)	2115.12 +/− 957.32
Endometrial thickness on HCG day(mm)	10.69 +/− 2.22
E2 on HCG day(pg/Ml)	3489.62 +/− 112.21
P on HCG day (ng/mL)	0.62 +/− 0.48
LH on HCG day ( IU/L)	1.031 +/− 1.04
No. of oocyte retrieved	12.96 +/− 5.82
No. of oocyte fertilized	8.43 +/− 3.88
No. of embryos	7.33 +/− 4.13
No. of high quality embryos	5.21 +/− 3.33
No. of embryos transferred	2.12 +/− 0.40
Etiology of infertility	
Tubal factor	91.31%
Endometriosis	1.24%
PCOS	1.24%
Multiple factors	6.21%

In women with Patterns A and B, the pregnancy rate were 55.2% and 50.9%, respectively, which was significantly higher than the rate of 37.4% in women with Pattern C (P < 0.05), while there was no difference between Patterns A and B (55.2% vs. 50.9%, respectively; P > 0.05). The implantation rates differed significantly between women with patterns A, B and C (35.3% vs. 32.1% vs. 23.4%, respectively; P < 0.05). Progesterone levels on the day of hCG administration among women with pattern C was significantly higher than those of women with Patterns A and B (Table 2).

**Table 2:** Clinical outcome by endometrial pattern and thickness

**Groups (n)**	**P on HCG day (ng/mol)**	**No. of embryos transplanted (n)**	**No. of clinical cycles (n)**	**No. of embryos implanted(n)**	**Clinical pregnancy rate(%)**	**Implantation rate (%)**
Pattern A (1094)	0.58 ± 0.41^ab^	2315	604	818	55.2^*△^	35.3^★▲^
Pattern B (684)	0.65 ± 0.53^bc^	1455	348	467	50.9^*○^	32.1^★◆^
Pattern C (155)	0.79 ± 0.65^ca^	333	58	78	37.4^△○^	23.4^▲◆^
Group 1 (47)	-	-	-	-	25.5^◊※^	13.0^●■^
Group 2 (1749)	-	-	-	-	52.1^◊☆^	33.8^●#^
Group 3 (137)	-	-	-	-	63.5^※☆^	39.1^■#^

Note: Pattern A was defined a triple-line pattern consisting of a central hyperechogenic line surrounded by two hypoechoic layers; Pattern B was defined an intermediate isoechogenic pattern with the same reflectivity as the surrounding myometrium and a poorly defined central echogenic line; Pattern C was defined as homogeneous, hyperechogenic endometrium. Group 1: endometrial thickness was ≤7 mm; Group 2: endometrial thickness was >7 mm to ≤14 mm; Group 3: endometrial thickness was>14 mm.

^△^P^○^P < 0.05; ^★^P^▲^P^◆^P < 0.05, ^a^P^b^P^c^P < 0.01; ^☆^P < 0.05, ^◊^P^※^P^●^P^■^P < 0.01. There is significant difference between the groups ( P < 0.05).

Clinical pregnancy rates were 25.5% in group 1 (≤7 mm), 52.1% in group 2 (>7 mm to ≤14 mm) and 63.5% in group 3 (> 14 mm), and the difference between the groups was statistically significant (P < 0.05) (Table 2). Implantation rate among group 3 was significantly higher than that of groups 1 and 2, and there was no significant difference between groups 1 and 2. Endometrial thickness was further evaluated at threshold increments of 1 mm to assess its discriminatory ability for clinical pregnancy. Pregnancy rates ranged from 28.6% among patients with an endometrial thickness of ≤6 mm to 67.7% among patients with an endometrial thickness of >16 mm. Implantation rates also increased with increasing endometrial thickness (date not shown). An endometrial thickness threshold of 7 mm was observed below which pregnancy rates decreased rapidly.

For further analysis, the three endometrial thickness groups were subdivided into three endometrial pattern groups. In group 1, pregnancy rates and implantation rates showed no significant differences between those with patterns A, B and C (pregnancy rates: 27.8% vs. 20.8% vs. 40.0%, respectively; P >0.05; implantation rates: 15.8% vs. 9.6% vs. 20%, respectively; P > 0.05). Among group 2, the pregnancy rates and implantation rates were significantly different between groups A, B and C (pregnancy rates: 55.6% vs. 50.2% vs. 34.3%, respectively; P < 0.05; implantation rates: 35.7% vs. 31.9% vs. 22.1%, respectively; P < 0.05). In group 3, there was no difference in clinical pregnancy and implantation rates between women with the three patterns (pregnancy rates: 56.0% vs. 76.1% vs. 62.5%, respectively; implantation rates: 35.5% vs. 46.0% vs. 35.3%, respectively; P > 0.05). Clinical pregnancy and implantation rates increased significantly with increasing endometrial thickness only among those with pattern A, but showed no significant increase with endometrial thickness among those with patterns B and C. (Table 3).

**Table 3:** The relationship between clinical outcome and endometrial thickness and pattern

	**Clinical pregnancy rate (%)**				**Implantation rate (%)**			
***Group***	**Pattern A**	**Pattern B**	**Pattern C**	***P***	**Pattern A**	**Pattern B**	**Pattern C**	***P***
*Group 1*	27.8 (5/18)	20.8 (5/24)	40 (2/5)	NS	15.8 (6/38)	9.6 (5/52)	20 (2/10)	NS
*Group 2*	55.6 (557/1001)	50.2 (308/614)	34.3 (46/134)	<0.05	35.7 (757/2122)	31.9 (416/1303)	22.1 (64/289)	<0.05
*Group 3*	56.0 (42/75)	76.1 (35/46)	62.5 (10/16)	NS	35.5 (55/155)	46.0 (46/100)	35.3 (12/34)	NS
*P*	NS	<0.01	NS		<0.05	<0.01	NS	

Endometrial thickness: Group 1: ≤7 mm; Group 2: >7 mm to ≤14 mm; Group 3: >14 mm.

Pattern A was defined as a triple-line pattern consisting of a central hyperechoic line surrounded by two hypoechoic layers. Pattern B was defined as an intermediate isoechogenic pattern with the same reflectivity as the surrounding myometrium and a poorly defined central echogenic line. Pattern C was defined as homogenous, hyperechogenic endometrium.

NS: not significant

## Discussion

Some studies have reported a significant correlation between endometrial thickness and pregnancy rate [9,18-20]. However, some do not support this view [1,13]. Our results agreed with previous studies that reported a correlation between endometrial thickness and clinical pregnancy. This clear relationship provided additional evidence to suggest that endometrial thickness is a useful indicator of endometrial receptivity.

Many studies have found a thin endometrium to be associated with a lower implantation rate, but no absolute cutoff for endometrial thickness exists; good pregnancy rates have been reported in cycles with endometrium <6 mm, and a successful pregnancy has been reported with endometrial thickness of only 4 mm [17]. Noyes N et al. [8] found that clinical pregnancy rate and live birth rate were significantly lower when endometrial thickness was less than 8 mm than when endometrial thickness was ≥9 mm. In the present study, the thinnest endometrial lining for successful clinical pregnancy was 4.8 mm. The clinical pregnancy (25.5%) and implantation (13%) rate in group 1 was significantly lower than groups 2 and 3. The relatively lower pregnancy rate observed in this group suggests that more attention needs to be given to embryos transferred to such patients.

Why does a thinner endometrium result in implantation failure? Casper RF [21] speculated that it may be related to oxygen tension. When the thickness measured by ultrasound is < 7 mm, the functional layer is thin or absent, and the implanting embryo would be much closer to the spiral arteries and the higher vascularity and oxygen concentrations of the basal endometrium. The high oxygen concentrations near the basal layer could be detrimental compared with the usual low oxygen tension of the surface endometrium.

Weissman et al. [22] showed that pregnancy rate was significantly lower above a maximum thickness of 14 mm, and they also suggested a possible increase in spontaneous abortion rates. Rashidi et al. [11] reported no pregnancies with an endometrial thickness >12 mm (n = 9). However, Richter et al. [4] and Ai-Ghamdi et al. [23] demonstrated a significant increase in the pregnancy rates as endometrial thickness increased, which was independent of the number and quality of the embryos transferred. In the present study, implantation and pregnancy rate increased with increasing endometrial thickness. Therefore, our findings support some previous studies in which increased endometrial thickness did not have a detrimental effect on clinical outcome. A case report [24] has described a successful twin IVF pregnancy in a woman with an endometrial stripe measuring 20 mm. In our study, the maximum endometrial thickness for a successful pregnancy was 19.7 mm.

Ultrasound measurement of endometrial pattern has been suggested to predict pregnancy outcome, but consensus has not been reached regarding the importance of either variable. Some studies [10,25-27] believed that a trilaminar pattern of the endometrium was correlated with higher implantation and pregnancy rates, while other studies did not find a significant relationship between endometrial pattern and pregnancy rate [11,18,28,29].

Our analysis found that significantly decreased implantation and pregnancy rates were observed in women without a triple-line endometrial pattern on the day of hCG administration. Several studies have suggested that a premature secretory endometrial pattern is introduced by the advanced P rise, and this premature conversion has an adverse effect on pregnancy rates. In our study, higher P levels were found in women with patterns C and B compared to those with pattern A (0.79 ng/mol vs. >0.65 ng/mol vs. >0.58 ng/mol, respectively; P < 0.05). However, another team [30] found that Progesterone receptor-B has stimulatory effects and an increased PR-B expression induced by ovarian stimulation would lead to the persistence of a proliferative endometrium. The delayed endometrial maturation would thus be desynchronized with the stage of embryo development, leading to decreased implantation rates in ART cycles. The exact mechanism for this is not known, and a rational explanation for this phenomenon awaits further study.

Despite a lower pregnancy rate and implantation rate when a homogeneous, hyperechoic pattern is noted, we disagree with some investigators who recommend embryo cryopreservation and subsequent ET in a frozen cycle. We agree with Friedler [31] that endometrial pattern offers important predictive information but should not be used as an absolute predictor of conception. Therefore, we believe that such patients should be adequately counseled and given the most adaptive advice.

When assessing the combined effect of endometrial thickness and pattern on clinical outcome, we found that the clinical pregnancy and implantation rates were not significantly different between women with patterns A, B and C in group 1 ( P > 0.05), which may indicate that a thinner endometrium represents poor receptivity of the endometrium regardless of endometrial pattern, while Chen et al. [18] found that a thinner endometrial thickness with a triple line pattern is associated with a higher clinical pregnancy rate compared to a thinner endometrium with no triple line pattern. There was also no difference between the patterns in group 3, and perhaps adequate endometrial thickness (>14 mm) mitigated the detrimental impact of not having a triple line pattern. There was significant difference in clinical pregnancy and implantation rates between women with the three patterns in group 2. These findings were not in accord with previous studies. Check et al. [26] found that no pregnancies occurred in patients with homogeneous hyperechoic endometrium, and Chen et al. [18] found that there were no differences in clinical pregnancy rate between patterns when endometrial thickness was ≥7 mm. Our results suggest that endometrial pattern has an effect on pregnancy rate when women have a moderate endometrial thickness (7–14 mm).

There are several possible explanations for these inconsistencies. Most studies assessed endometrial thickness and pattern on the day of or following hCG administration and on the day of oocyte retrieval, while other studies assessed the endometrium on the day of ET, and even fewer assessed it on both the days of hCG injection and ET. Therefore, the optimal timing of endometrial assessment remains unknown. Previous studies found that assessment on the day of hCG might be more useful as a prognostic test given the earlier timing and the absence of P exposure [32,33].

In addition, it is necessary to note that the correlation between endometrial thickness and pattern and pregnancy outcome shown in our study does not imply a causal relationship. The relationship may merely result from some other factors that are directly responsible for endometrial receptivity (such as blood flow or some other underlying physiological machinery responsible for cyclic endometrial development). Therefore, although some treatments may significantly improve endometrial thickness, such therapies may not necessarily have any clinical benefit in terms of pregnancy rate.

This study has some limitation, the most important of which is that it is retrospective in nature. However, we believe the results are of interest because similar studies have published with conflicting results. A well-designed and powered randomized clinical trial will be needed to confirm this result.

## Conclusions

When endometrium thickness was ≤ 7 mm, other prognostic factors, such as embryo quality and age, should be taken into consideration. Because endometrial thickness of ≤ 7 mm was observed in only 2.4% of cycles in our study, further study is needed to make a definitive conclusion regarding this group. Regardless of the endometrial pattern, a thicker endometrium (>14 mm) did not have an adverse effect on the clinical outcome. Endometrial pattern can be considered when women have a moderate endometrial thickness.

## Competing interests

The authors declare that they have no competing interests.

## Authors’ contributions

Zhao J carried out the acquisition of data, analysis and interpretation of data and writing of the manuscript. Zhang Q has been involved in the ultrasound examination and in revising the manuscript. Li YP conceived of the study, participated in its design and coordination, and helped to draft the manuscript. All authors have read and approved the final version of the manuscript.
